# Trouble spots in literary conversation learning: managing bilinguals’ cognitive expectation problems using conversational presupposition theory

**DOI:** 10.1186/s40359-025-03473-7

**Published:** 2025-10-02

**Authors:** Yuguo Ke, Liang Chen, Xiaozhen Zhou

**Affiliations:** 1 Taizhou University, The City of Taizhou, 380000 Zhejiang province China; 2 Fudan University, Shanghai, China

**Keywords:** Cognitive expectation, Literary conversation learning, Conversational presupposition, Bilinguals

## Abstract

**Background:**

The significance of addressing bilinguals’ cognitive expectations in conversation is well established. However, the interplay between conversational presuppositions and cognitive expectations in bilingual contexts-particularly in literary settings—remains under-explored.

**Materials and methods:**

This cross-sectional study examines how bilinguals negotiate literary conversations in relation to both L1 and L2 cognitive expectations. Through a theoretical and empirical lens, we identify two distinct pathways by which conversational presuppositions operate in bilingual discourse.

**Results:**

These pathways prove critical for facilitating effective communication in bilingual learning environments. The study underscores the constructive role of cognitive expectations in literary conversation analysis, framed within conversational presupposition theory. Our findings demonstrate that conversational expectations align with a comprehension framework capable of mitigating challenges posed by bilingual cognitive divergence. By extending this framework, we reveal how presupposition mechanisms can resolve issues tied to bilingual cognition, introducing a novel paradigm for enhancing multilingual learners’ understanding of diverse conversational structures.

**Conclusion:**

This research provides new insights into how presuppositions facilitate language comprehension and deepens our understanding of literary conversational dynamics. Ultimately, it advances communicative coherence in bilingual contexts and offers a fresh perspective on optimizing cross-linguistic dialogue.

## Background

The theoretical framework of “conversational presupposition” traces its philosophical roots back to the Frege-Strawson tradition of presupposition theory, especially in the analysis of definite descriptions [1–3]. It is increasingly recognized that the seminal work of [1] in On Sense and Reference first laid the groundwork for key concepts such as presupposition triggering and acceptance. Conversational presupposition denotes an implicit assumption or background information that a speaker assumes to be shared knowledge with the listener, thereby ensuring the coherence and meaningfulness of the utterance [1]. These presuppositions emerge from the interaction context and the participants’ shared beliefs, cultural knowledge, or prior discourse. Historically, presupposition research has been acknowledged as a significant factor in semantic analysis and conversational contexts, yet it has largely neglected cognitive dimensions [4–6]. Although subsequent scholars [7–11] have notably broadened the scope of presupposition studies, there remains a surprising lack of development in the literature regarding the cognitive underpinnings of conversational presupposition, an area that has garnered particular attention [12–15]. The conversational presupposition theory posits that this cognitive gap constitutes a critical theoretical limitation, especially in understanding how interlocutors achieve mutual comprehension during bilingual conversation learning through the mechanisms of conversational implicature.

It is posited that contemporary research on conversational presupposition plays a pivotal role in two analytical trajectories and is thus crucial for fostering optimal cognitive development in conversation learning. Aligned with this theory, findings indicate that literary conversation learning styles are linked to more favorable expected outcomes for bilinguals experiencing both typical [6, 12, 13] and atypical development [14, 15] in cognitive expectation issues.

The first trajectory, exemplified by studies [16–22], employs a micro-linguistic approach, concentrating on sentence-level semantics and lexical meanings. Within the realm of literary conversations, enhancing the quality of bilinguals’ cognitive expectations has emerged as a primary objective in preemptive cognitive-mediated interventions. These interventions aim to support more optimal developmental outcomes for bilinguals who are at high risk of cognitive expectation problems. In this context, it is not posited that literary conversations are the ‘cause’ of cognitive expectation problems; rather, it is suggested that atypical literary cues encountered early in learning may influence bilinguals’ learning styles, thereby altering the quality of literary input they receive.

While this tradition has provided valuable insights into local linguistic structures, it falls short in accounting for the dynamic cognitive processes that underlie real-time presuppositional interpretation [13]. Trouble spots in literary conversation denote instances in dialogues where miscommunication, ambiguity, or cognitive dissonance emerge due to unshared presuppositions (e.g., cultural references, private knowledge). The second trajectory acknowledges that the quality of presupposition transcends purely semantic or structural considerations, being fundamentally influenced by cognitive expectations concerning conversational identity. Although these findings hold promise for improving the quality of literary learning and reducing the severity of cognitive expectation issues, these studies generally overlook critical factors, such as conversational presuppositions, that may impact bilingual processing of presupposition in literature and consequently enhance language learning methodologies. Research thus far has identified associations across two key domains: presuppositions across languages and cultures, including cognitive expectational behaviors and misunderstandings or implicit assumptions in multicultural settings. This work will be elaborated upon in the following sections. However, no studies have explored models that link all these domains together.

Given the associations between these domains, it is imperative to investigate potential causal relationships, which may facilitate the prioritization of cognitive expectation considerations in interventions specifically tailored to meet the needs of bilinguals and ensure optimal outcomes. Considering that many early interventions rely on conversational presuppositions to deliver the intervention, it is essential to support bilinguals by fostering their strengths and providing targeted assistance in areas where they may encounter difficulties. From this perspective, we argue that conversational implicature—when appropriately contextualized within cognitive expectations—requires analysis through an integrated semantic-presuppositional framework [23]. The current overemphasis on decontextualized linguistic analysis risks overlooking the essential cognitive underpinnings of communicative effectiveness.

One strand of research has revealed that literary conversation learning triggers a distinctive cognitive expectation process, marked by a psychological interplay between semantic comprehension and mutual anticipatory mechanisms [24–26]. Research indicates that this intersection remains theoretically underexplored within traditional presupposition theory, which has yet to sufficiently elucidate: (1) the precise pathways through which literary conversation learning fosters this cognitive-semantic interface, and (2) how cognitive expectation factors quantitatively and qualitatively shape conversational outcomes.

Previous studies may have neglected how bilinguals reconcile conflicting presuppositions arising from their languages or how they transfer presuppositional strategies across linguistic boundaries [24]. Alternatively, they may have predominantly focused on monolingual speakers, assuming uniform processing patterns [25], or often treated bilinguals as a homogeneous entity, overlooking how individual differences influence presupposition processing [26]. Furthermore, some research may have limited applicability to real-world scenarios where bilingualism is prevalent rather than exceptional [27]. However, bilinguals’ unique cognitive architecture may give rise to distinct strategies for interpreting presuppositions, particularly in ambiguous or culturally charged contexts. These theoretical gaps highlight the pressing need for a cognitive-expectation model of conversational presupposition that could substantially propel research on conversational implicature forward.

These findings provide some statistical evidence that the practical implications of these theoretical limitations are profound. Many observed communicative breakdowns—particularly social conflicts in everyday conversations—can be attributed to poorly managed cognitive expectations within presuppositional contexts. This reality underscores the necessity of developing a more robust theoretical paradigm that systematically integrates cognitive expectation mechanisms with established presupposition theory. Such an integrated framework would not only deepen our theoretical understanding of conversational dynamics but also furnish practical tools for enhancing cross-linguistic and cross-cultural communication. Therefore, it appears that higher levels of cognitive challenges significantly affect bilinguals’ cognitive expectation behaviors in literary learning.

### The present study

The objective of the present study was to identify and address trouble spots in literary conversation learning among bilinguals, specifically focusing on managing their cognitive expectation problems through the lens of conversational presupposition theory. While prior research has often centered on systematically investigating conversational presupposition theory by examining cognitive expectations—analyzing both their enabling (affirmative) and limiting (restrictive) roles, as well as their structural representations across cognitive and non-cognitive domains—our approach builds upon Yule’s presupposition theory to develop an integrated analytical framework. This framework aims to clarify the mechanisms underlying conversational generation, supported by empirical case studies that illustrate how presuppositional phenomena manifest across various types of expectations. The bilingual participants in this study were recruited from middle schools, a demographic likely to exhibit a wider range of developmental concerns, including cognitive behaviors indicative of expectation-related issues.

Drawing on previous findings, we anticipated that while existing research has primarily explored bilingual identity negotiation within constrained contexts, our study would pioneer new territory by examining the dynamic, three-way relationship between language, cognition, and interaction within literary discourse [27]. This, in turn, could have far-reaching effects on cognitive expectation problems, with literary learning being a significant component. Moreover, we predicted that the strategic integration of conversational presupposition theory with comprehension mechanisms would lead to three notable advancements: (1) enhanced cognitive-conversational alignment, (2) improved literary dialogue generation, and (3) resolution of persistent challenges in presuppositional communication. These advancements build upon [14]’s foundational identification of two distinct pathways for literary conversation generation—cognitive versus non-cognitive expectations.

Therefore, we hypothesized that our bilingual cognitive expectation paradigm would offer fresh insights into three critical areas: the activation mechanisms of literary conversations, the cognitive architecture underlying presuppositional understanding, and the optimization of cross-linguistic communicative coherence. The proposed framework not only introduces an innovative methodology for analyzing conversational dynamics but also significantly boosts the practical applications of implicature research in real-world settings. Collectively, these findings highlight the urgent need to revise existing presupposition theory to better incorporate cognitive expectation mechanisms, thereby advancing both theoretical models and their practical implementation in literary conversation generation systems.

## Methods

### Participants

A total of 300 bilingual adolescents proficient in both Chinese and English took part in the Mechanism of Bilingual Development Study (MBDS), which was carried out across three distinct cities in China: Shanghai, Taizhou (located in Zhejiang Province), and Jingdezhen (in Jiangxi Province). These cities were deliberately chosen for their varied bilingual proficiency profiles, especially within educational settings. The MBDS utilized a single-blind randomized controlled trial (RCT) design to assess the effectiveness of interventions aimed at addressing difficulties in literary conversational learning among bilinguals aged between 13 and 18 years who exhibited cognitive expectation deficits. Recruitment strategies were tailored to the proficiency levels and specific circumstances of each city. In Shanghai, 100 participants with advanced English proficiency were identified through referrals from a specialized bilingual development school, all of whom showed delays in cognitive expectations. Similarly, in Taizhou, 100 intermediate-level English learners were recruited through local educational referrals, while in Jingdezhen, 100 participants with lower English proficiency were referred directly by their schools. All participants underwent a screening process using the Social Attention and Communication Surveillance-Revised (SACS-R) checklist [28–30], a well-validated tool designed to detect cognitive challenges during bilingual development. Due to the diverse recruitment methods employed, trained study personnel conducted a standardized eligibility assessment. To be eligible, participants were required to demonstrate cognitive deficits in at least three out of five key behaviors outlined in the SACS-R. These behaviors assess phonological, morphological, syntactic, and lexical aspects of literary conversation learning and are indicative of cognitive expectation problems [7]. The SACS-R facilitates routine bilingual health checks by requiring specific cognitive responses for each item, which are categorized as either “cognitive” or “cognitively impaired.” For example, the pragmatics subscale poses questions such as, “Do you understand this? Use facial expressions, observe body language, and recognize figurative meaning.” The tool boasts robust diagnostic validity, with an estimated positive predictive value of 81.6% for identifying cognitive issues [16].

### Data collection

This study draws on the data collected by examining bilingual conversational learning as a core component of a broader investigation into language practices among 300 Chinese-English bilingual middle school students. The difficulty level of the texts (assuming they are academic discussions on presuppositions in literary analysis, as previously outlined) have been evaluated along several dimensions, including linguistic complexity, conceptual abstraction, interdisciplinary demands, and target audience. Authors have adjusted the necessary prompts to guarantee all the participant can know the meaning of the literary conversation.

This study leverages data gathered through an in-depth examination of bilingual conversational learning, a pivotal element of a broader investigation into language practices among 300 Chinese-English bilingual middle school students. The texts under scrutiny, presumed to be academic discussions on presuppositions in literary analysis as previously described, were evaluated based on several criteria, including linguistic complexity, conceptual abstraction, interdisciplinary demands, and the intended audience. Authors meticulously refined the prompts to ensure that all participants could comprehend the meaning of the literary conversations.

The analysis utilizes baseline data from the Mechanism of Bilingual Development Study (MBDS), a randomized controlled trial (RCT) executed within five weeks following eligibility screening. Participants filled out self-report questionnaires and underwent behavioral assessments across four schools located in Shanghai, Taizhou, and Nanchang. All evaluations were conducted by blinded study personnel to mitigate potential bias. The study focused on ten literary conversations extracted from globally acclaimed literary works: New Year’s Sacrifice by Lu Xun, Xiao Erhei Gets Married by Zhao Shuli, Rickshaw Boy by Lao She, Border Town by Shen Congwen, Thunderstorm by Cao Yu, Wuthering Heights by Emily Brontë, Jane Eyre by Charlotte Brontë, A Tale of Two Cities by Dickens, Moby-Dick by Melville, and Pride and Prejudice by Austen. These texts were selected for their linguistic richness and cultural significance to bilingual development.

Each of the 300 participants underwent thorough cognitive assessments to evaluate developmental outcomes, completed standardized self-report measures, and participated in structured cognitive activities aimed at capturing the processes involved in literary conversational learning. These activities were systematically coded to analyze both linguistic and cognitive engagement. To investigate the relationships between participant characteristics and conversational learning outcomes, a multidimensional dataset was compiled, encompassing demographic, linguistic, and cognitive variables. The final sample consisted of 300 dyadic interactions, with detailed participant profiles and methodological procedures elaborated in subsequent sections.

### Measures

#### Conversational presupposition

Conversational presupposition centers on the implicit assumptions or background information that a speaker assumes to be shared knowledge with the listener, thereby ensuring that the utterance is coherent and meaningful. These presuppositions emerge from the context of the interaction, as well as from the participants’ shared beliefs, cultural knowledge, or prior discourse. To identify conversational presuppositions in literary dialogues or real-life conversations, researchers employ the following methods: (1) Factive Verbs: Verbs such as “realize,” “know,” “regret,” and “forget” presuppose the truth of the embedded clause. For example, in the sentence “She realized that it was raining,” the verb “realized” presupposes that it is indeed raining. (2) Cleft Sentences: Structures like “It was John who called” presuppose that someone made a call. This grammatical construction emphasizes a particular element while presupposing the occurrence of the event itself. (3)Implicature and Indirect Speech Acts: Statements such as “Can you pass the salt?” presuppose not only that passing the salt is possible but also that it is relevant in the given context. This indirect approach relies on shared understanding to convey meaning beyond the literal words. (4) Contextual and Cultural Analysis of Shared Background Knowledge: This involves identifying references to historical events, cultural norms, or interpersonal relationships that the characters (or speakers) are assumed to know. For instance, a literary dialogue might allude to a well-known historical event, presupposing that the audience understands its significance and relevance.)

#### Trouble spots in literary conversation

Trouble spots in literary conversation refer to those moments in dialogues where miscommunication, ambiguity, or cognitive dissonance occur due to unshared presuppositions—such as cultural references or private knowledge—as well as linguistic complexity (e.g., idiomatic expressions, metaphors), emotional or social tension (e.g., sarcasm, indirect criticism), and bilingual or cross-cultural mismatches. These trouble spots are crucial for analyzing how readers or speakers navigate meaning and resolve confusion. To investigate how students engage with these trouble spots in literary conversations, researchers have employed the following methods: (1) Close Reading and Annotation Identification Tasks: For instance, students are asked to highlight passages where they find confusing or ambiguous dialogue. This helps pinpoint specific areas where miscommunication may arise. (2)Presupposition Mapping: Students are tasked with noting implied assumptions and discussing whether these assumptions are shared by all characters. An example prompt might be: “In this scene, why does Character A seem surprised by Character B’s response? What unstated belief or fact does Character B assume that Character A doesn’t know?” This encourages students to delve into the underlying presuppositions that shape the dialogue. (3)Role-Play and Reenactment: Acting Out Ambiguities: Students perform dialogues with intentional misinterpretations to explore how presuppositions affect meaning. This interactive approach allows them to experience firsthand how miscommunication can occur. (4)Alternative Endings: Students rewrite conversations where trouble spots are resolved differently, such as through explicit clarification versus continued misunderstanding. An example activity could be: “Rewrite this exchange so that Character A explicitly states their presupposition. How does this change the tone and outcome of the conversation?” This encourages creative thinking and a deeper understanding of how presuppositions influence dialogue. (5)Group Discussion and Debate: Perspective-Taking: Students debate why characters might have different interpretations of the same dialogue, fostering critical thinking and empathy. Additionally, cultural context analysis can be incorporated to discuss how presuppositions differ across cultures, enhancing students’ cross-cultural awareness.

By defining conversational presupposition as implicit shared knowledge and trouble spots as moments of miscommunication, researchers have operationalized their study through linguistic analysis, contextual interpretation, and student-centered activities. These constructs help illuminate how meaning is negotiated in literature and, in particular, how bilingual readers navigate complex linguistic and cultural landscapes.

## Results

The theory of conversational presupposition, as extensively examined by scholars such as those cited in [31–35], offers a nuanced exploration of bilingual communication through the lens of conversational presupposition. This theoretical framework transcends the conventional linear analysis, which tends to concentrate on isolated sentences, by integrating cognitive paradigms related to conversational identity. Furthermore, it expands the horizons of contextual cooperation inherent in conversational presuppositions, thereby providing a more comprehensive understanding of how bilingual speakers navigate and construct meaning within dialogic interactions.

### Bilinguals’ trouble spots during literary conversational learning

By integrating conversational presupposition with cognitive processes, this theoretical framework offers a profound insight into the intricate interplay between cognitive and non-cognitive expectations in bilingual conversational learning (refer to Table 1). Within the purview of conversational presupposition, it pinpoints pivotal cognitive expectation tendencies—such as expectation intensity, distance, and the communicative context—that significantly mold linguistic interactions [14, 23]. Employing literary conversations as a methodological tool, this approach further unravels the interpersonal functions of discourse, thereby enhancing both linguistic proficiency and communicative effectiveness through subtle expectation management.

**Table 1:** Trouble spots of cognitive expectations of bilinguals: types, frequencies, and examples

Type of conversational presuppositions	frequency	Examples
expectation cognition	196	Extract (1) 1 Village Chief (clear-headed, persuading Xingwang): “Xiao Erhei indeed contracted malaria, it’s not feigned illness. As for falling in love with someone, it’s not illegal, we can’t tie people up.” 2 Xingwang said: “He already has a woman.” 3 Village Chief said: “Everyone in the village knows Xiao Erhei doesn’t acknowledge his bilingual bride. It’s right for them not to acknowledge it.” (Lu Xun, “New Year’s Sacrifice”)
expectation intensity	168	Extract (2) 1 Village Chief (clear-headed, persuading Xingwang): “Xiao Erhei indeed contracted malaria, it’s not feigned illness. As for falling in love with someone, it’s not illegal, we can’t tie people up.” 2 Xingwang said: “He already has a woman.” 3 Village Chief said: “Everyone in the village knows Xiao Erhei doesn’t acknowledge his bilingual bride. It’s right for them not to acknowledge it.” (Zhao Shuli, “Xiao Erhei Gets Married”)
expectation distance	181	Extract (3) 1 Xiaoma (nodded towards the steamed bun, sniffed once): “Grandpa, have three of them, the rest are mine. I’ll take you home later!” 2 The old man (smiling proudly at everyone): “No need, later we’ll still walk, sitting in the car is too cold!” (Lao She, “Rickshaw Boy”)
expectation orientation	298	Extract (4) 1 Healthcliff: “I must go, Katy, but if I’m still alive, I’ll come and see you before you go to bed. I will not go five yards from your window. 2 Katy: You must not go! I tell you, you mustn’t go. 3 Healthcliff: only an hour. 4 Katy: not a minute. 5 Healthcliff: I must go, as if Linton is coming… (Emily Brontë’s Wuthering Heights)
expectation confusion	272	Extract (5) Jane Eyre and Rochester are discussing Mr. Mason.) 1 Rochester: Still up? 2 Jane Eyre: How can I sleep until I see you back safely? How’s Mr. Mason? 3 Rochester: He’s fine. With a doctor looking after him. 4 Jane Eyre: Did you get over the danger you said you were in last night? 5 Rochester: There is no guarantee that Mason will not leave the UK. The sooner the better. 6 Jane Eyre: He doesn’t look like a man who wants to hurt you. (Charlotte Brontë’s ‘Jane Eyre)

For instance, in Extract (1), the presupposition associated with “Mrs. Shiang Lin” unveils cognitive discrepancies between the narrator (“me”) and the short worker, illustrating how divergent expectations extend the analysis of presupposition beyond mere semantic connotations. Similarly, the delicate balance between expectation and non-expectation in the induction and acceptance of presuppositions emerges as crucial for conveying conversational implicature. In Extract (2), the cognitive interpretations of Xiao Erhei’s actions by the village chief and Xingwang, coupled with their respective presuppositions, create a non-cognitive expectation dynamic between the speaker and listener. Meanwhile, Extract (3) exemplifies cognitive non-expectation in a dialogue between Xiaoma and an elderly interlocutor, where the speaker’s anticipated response is met with an unexpected rejoinder, underscoring the pivotal role of mismatched expectations in shaping presuppositional outcomes. These examples collectively emphasize the bidirectional influence of cognitive and non-cognitive expectations on conversational presupposition.

Furthermore, the theory integrates perspectives on communicative utility and dynamic identity selection, highlighting how identity construction mediates communicative needs and linguistic choices [11, 36]. Consequently, a thorough analysis of speakers’ presuppositions and their conversational expectations becomes indispensable for comprehending the presuppositional mechanisms inherent in situated bilingual discourse.

Table 1 demonstrates that the main context of five extracts are: (1) The Village Chief, being clear - minded, tries to persuade Xingwang. He states that Xiao Erhei genuinely has malaria, not pretending to be ill. Regarding falling in love, it’s not against the law, so people shouldn’t be restricted. Xingwang counters that Xiao Erhei already has a woman. The Village Chief responds that the whole village knows Xiao Erhei doesn’t recognize his bilingual bride, so it’s reasonable they don’t accept it. (2) The Village Chief, with a clear - thinking mind, attempts to persuade Xingwang. He emphasizes that Xiao Erhei truly has malaria, not faking it. He also points out that falling in love isn’t illegal, so people shouldn’t be forcibly controlled. Xingwang retorts that Xiao Erhei already has a partner. The Village Chief then says that the whole village is aware Xiao Erhei doesn’t recognize his bilingual bride, so it’s justifiable for them not to accept it. (3)In this extract from Lao She’s Rickshaw Boy, Xiaoma shows consideration for the old man. He nods at the steamed buns, takes a sniff, and offers three to his grandfather, claiming the rest for himself. He also promises to take the old man home later. However, the old man, with a proud smile to everyone around, declines the offer of a ride, saying they’ll walk instead as he finds sitting in the car too cold.(4) In this extract, Heathcliff informs Katy of his intention to leave but promises to visit her before she goes to bed and stay close to her window. Katy strongly objects, pleading with him not to go, not even for a minute. However, Heathcliff remains firm, stating he has to leave, hinting at Linton’s impending arrival as a reason. The conversation reveals Katy’s reluctance to part with Heathcliff and his determination despite her protests.(5)In this dialogue from Jane Eyre, Rochester and Jane discuss Mr. Mason. Rochester is surprised Jane is still awake, and she explains she can’t sleep until he returns safely, then inquires about Mason’s condition. Rochester assures her Mason is fine with a doctor’s care. Jane asks if Rochester has overcome the danger he mentioned before. Rochester implies Mason might leave the UK soon for safety. Jane notes Mason doesn’t seem like a threat to Rochester.

Table 1 demonstrates that the formation of conversational presuppositions is influenced by distinct psychological tendencies, notably affirmation and restriction [12]. As Talmy (2018, p. 93) notes, “the development of conversational presupposition tendencies presupposes an implicit community; deviations from this norm trigger cognitive expectation or non-expectation responses.” This highlights the importance of examining both explicit and implicit psychological expectation frameworks in literary conversational learning, encompassing aspects such as expectation cognition, intensity, distance, orientation, and ambiguity. Such exploration should address presuppositional patterns associated with affirmation, restriction, cognitive expectation, and non-cognitive expectation.

In literary conversational learning, presupposition parameters unveil subtle cognitive expectation tendencies, including intensity, contextual relevance, and interpersonal distance. The listener’s identity significantly shapes their expectations, motivations, needs, and emotional engagement. For instance, in Extract (4), Heathcliff, as a focal point of conversational presupposition, expresses expectation intensity through his declaration, “I will not go five yards from your window.” Kate’s response recalibrates this expectation, broadening its interpretive scope. Their interaction illustrates divergent cognitive presuppositions: Heathcliff anticipates departure, while Kate assumes retention, creating a dynamic of cognitive non-expectation. Heathcliff seeks permission to leave, whereas Kate expects him to stay, resulting in unmet expectations. As [14] contends, cognitive psychological expectations typically rely on lexical, encyclopedic, and logical frameworks, modulated by the listener’s identity and conversational intensity. Building on this [19], suggests that cognitive expectations underpin “knowledge scripts,” “psychological schemas,” and “social psychological representations,” delineating specific pathways within conversational presupposition.

Extract (4) serves as a prime illustration of the stark expectation gap between Heathcliff and Kate, underscoring the presence of unfulfilled anticipations within their discourse. This interaction sheds light on the semantic evolution that unfolds in dialogue, where Heathcliff reacts to external stimuli while relying on Kate to decipher not only the explicit meanings but also the underlying implicatures. Collectively, Extracts (1) through (4) provide valuable insights into the generation of presuppositions in the context of literary conversational learning, emphasizing the pivotal roles of cognitive expectation, non-expectation, affirmation, and restriction.

The real-time diversification of conversational settings serves as a conduit for transmitting information to the listener’s perceptual domain, where it undergoes processing through the reconstruction, recognition, and contrast of conversational symbols. This process reflects a remarkable degree of expectation flexibility [37], reminiscent of the cognitive expectation dynamics observed between “me” and the short worker in Extract (1).

As depicted in Table 1, the transmission from speaker to listener involves navigating between cognitive expectation and non-expectation pathways. However, the process of conversational presupposition along these routes lacks a clearly defined spatial categorization of expectation shifts. Cognitive mechanisms play a crucial role in constructing comprehension pathways for discourse by integrating extralinguistic content and contextual inference models. Nevertheless, a comprehensive framework for interpreting presuppositions in literary conversational learning remains elusive, potentially attributable to factors such as the listener’s capacity for expectation, motivations, needs, and emotional cues.

 [38] underscores that cognitive expectation internalizes events, modalities, or behaviors within the realm of literary conversational learning, stemming from the listener’s preexisting impressions and the speaker’s cognitive framing. Traditional conversational norms and the listener’s self-expectation needs serve to define presuppositional parameters, with cognitive expectation reflecting shared psychological tendencies. The speaker’s processes of attribution and interpretation relative to the listener further elucidate this intricate interplay. Consequently [31], argues that cognitive expectation constitutes the directional framework of conversational presupposition, shaping the patterns of psychological interaction. Therefore, further research is imperative to unravel the intricate mechanisms underlying conversational presupposition that are grounded in cognitive expectations, thereby enhancing our understanding of this fundamental aspect of literary discourse.

### Types of adjustments bilinguals made in response to cognitive expectation issues under the conversational presupposition paradigm

The Conversational Presupposition Theory (CPT) [39] provides a rigorous and insightful framework that places cognitive expectations at the forefront, shedding light on how bilingual conversational learning evolves from broad, generalized cognitive orientations to increasingly nuanced and refined mutual understandings. Given the vast diversity among literary texts in terms of genre, style, historical backdrop, and cultural presuppositions, the functioning of presuppositions is inevitably influenced by these factors. It is crucial for literary analysis to avoid the pitfall of overgeneralization when it comes to conversations within texts (for instance, not assuming that all literary presuppositions operate in the same manner as those in everyday conversational contexts). Instead, it should embrace the nuanced variations that exist, such as the distinct ways in which Victorian novels and modernist poetry encode assumptions. Additionally, the author’s intent must be taken into account, as a postmodern writer, for example, might deliberately subvert traditional presuppositions to achieve ironic effects. At the heart of CPT lies the pivotal role of semantic integration in molding conversational presuppositions. This process is mediated through several key mechanisms: trace conditioning, which involves the associative linking of linguistic cues to their contextual meanings; bidirectional processing, which refers to the reciprocal interaction between bottom-up perceptual analysis and top-down inferential reasoning; dynamic attention allocation, which is the adaptive focusing of cognitive resources on the most salient elements of discourse; temporal orientation, which encompasses the negotiation of past, present, and future referents within dialogue; and updating strategies, which involve the continuous revision of presuppositional frameworks in response to the influx of new information. Collectively, these mechanisms necessitate progressively more advanced cognitive competencies from the reader or listener (see Table 2).

**Table 2:** Pearson correlations between bilingual outcome variables and conversational presuppositions

types	trace conditioning	bidirectional processing	dynamic attention	temporal orientation	updating strategies
**Shanghai bilinguals**					
Positive bilingual learning	0.641	0.817*	0.715*	0.678	0.742*
Negative bilingual learning	0.414	0.595	0.342	0.347	0.383
**Taizhou bilinguals**					
Positive bilingual learning	0.577	0.618	0.705*	0.634	0.708*
Negative bilingual learning	0.316	0.346	0.324	0.417	0.523
**Jingdezhen bilinguals**					
Positive bilingual learning	0.491	0.527	0.466	0.514	0.645
Negative bilingual learning	0.224	0.292	0.197	0.318	0.344

As presented in Table 2, the functional distribution of presuppositions demonstrates considerable variability across different bilingual groups, with specific values noted as follows (e.g., 0.641, 0.817, 0.715, 0.678, 0.742; 0.577, 0.618, 0.705, 0.634, 0.708*; 0.491, 0.527, 0.466, 0.514, 0.645). Notably, bilinguals in Shanghai exhibit a significantly stronger alignment with the cognitive expectation dimension of conversational presuppositions when compared to their peers in Taizhou and Jingdezhen. Additionally, learners who have had positive bilingual acquisition experiences demonstrate enhanced perceptual acuity in discerning cognitive expectations embedded within conversational presuppositions. These observations lead us to conceptualize the formative process as a Cognitive Expectation Process (CEP), marked by the activation of anticipatory schemas, while the updating process aligns with a Cognitive Non-Expectation Process (DNP), which entails the adaptive revision of presuppositional frameworks. Although this study primarily focuses on cognitive mechanisms due to spatial constraints, future research endeavors will delve deeper into the dynamics of DNP, particularly examining the interplay between expectation violation and semantic realignment.

**Fig. 1:**
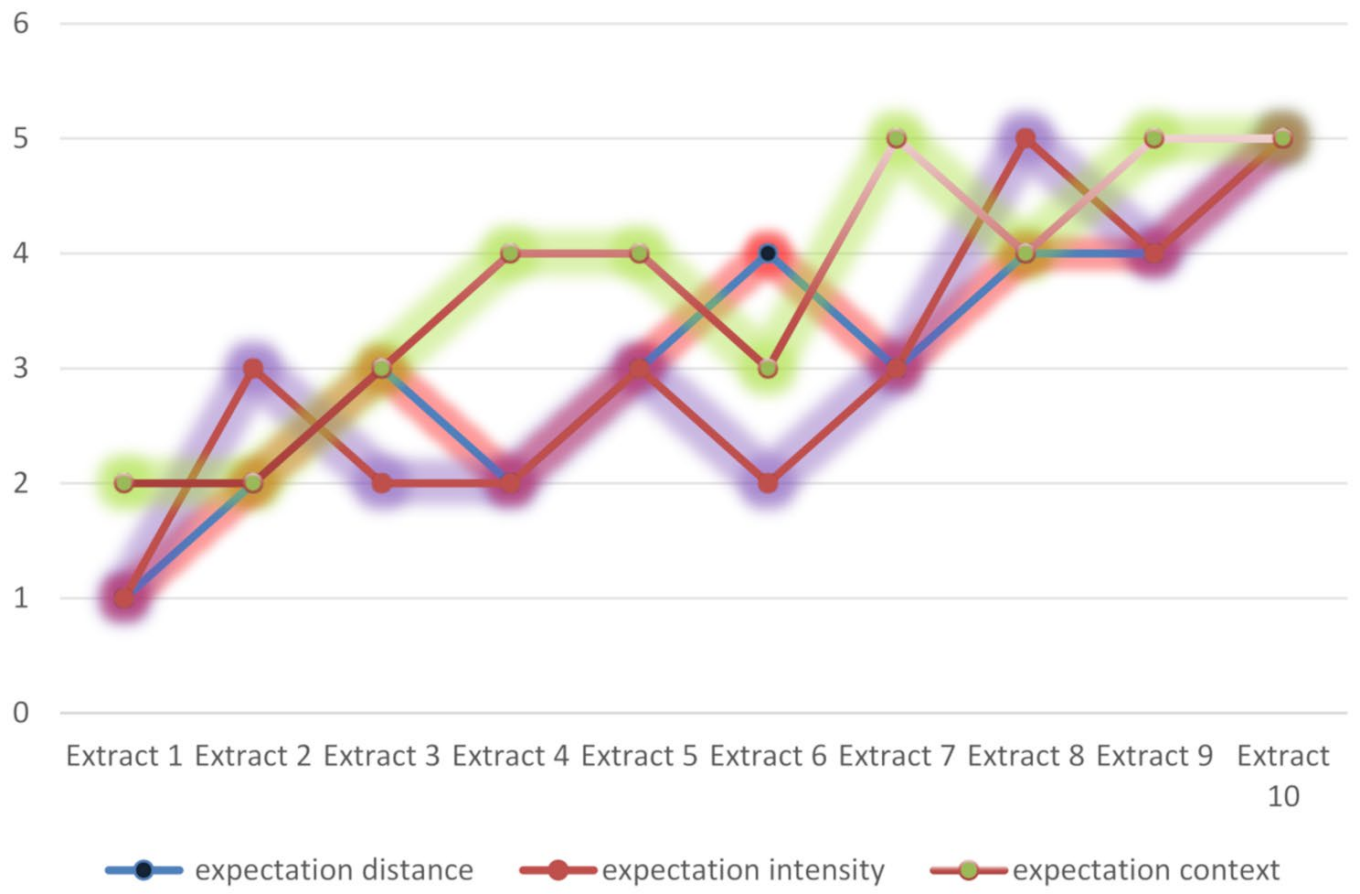
Cognitive Expectation Reports From Bilinguals

As shown in Fig. 1, bilingual participants evaluated three cognitive variables—expectation distance (the perceived discrepancy between anticipated and actual outcomes), expectation intensity (the degree of cognitive commitment to presuppositions), and expectation context (the situational framing of inferential processes)—via structured self-reports. These evaluations uncover statistically significant causal relationships among the variables, as supported by ascending correlation trends observed in cognitive semantics literature, thereby defining core cognitive categories in literary conversational processing. For instance, in Extract (5), when Rochester and Jane Eyre discuss “Mr. Mason’s potential harm to Rochester,” their presuppositional divergence serves as a prime illustration of the theory: Rochester assumes Mr. Mason presents an imminent threat, whereas Jane Eyre holds a contrary view. This asymmetry creates a cognitive non-expectation gap, with Rochester’s expectations centered on Jane Eyre’s responsive behavior. While Rochester anticipates Mr. Mason’s immediate departure from London, Jane Eyre’s counter-expectation results in an unmet discourse outcome, demonstrating how divergent cognitive frameworks shape conversational trajectories and the negotiation of mutual comprehension. Rochester’s query, “Still up?“, reflects a keen awareness of Jane Eyre’s discourse identity, adhering to CEP principles by establishing interactional presuppositions. Jane Eyre’s response, “How can I sleep until I see you back safely?“, further underscores the bidirectional influence of cognitive expectations on discourse structuring, revealing how presuppositions function as dynamic cognitive tools rather than static linguistic markers.

The semantic integration of conversational presuppositions, with Mr. Mason serving as a pivotal discourse anchor, systematically facilitates the construction of anticipatory cognitive frameworks. This intricate process is orchestrated by four interconnected mechanisms: trace conditioning, as exemplified by the query “How’s Mr. Mason?“, which forges associative links between lexical cues and contextual inferences; bidirectional processing, evident in exchanges like “He’s fine. With a doctor looking after him.” versus “He doesn’t seem like a man who wants to hurt you.“, which enables reciprocal validation of presuppositions through syntactic-semantic alignment; dynamic attention allocation, illustrated by “Did you get over the danger you mentioned last night?“, which prioritizes salient discourse elements to refine expectations; and temporal orientation, as demonstrated by “The sooner, the better.“, which anchors presuppositions within situational timeframes to enhance predictive accuracy. Bilingual learners who have had positive language acquisition experiences exhibit superior proficiency in harnessing these mechanisms, enabling them to navigate updating processes (adaptive revision of presuppositional frameworks), communicative strategies (alignment of discourse goals), and temporal repositioning (recontextualization of expectations across conversational phases) more effectively than their peers with negative experiences.

**Fig. 2:**
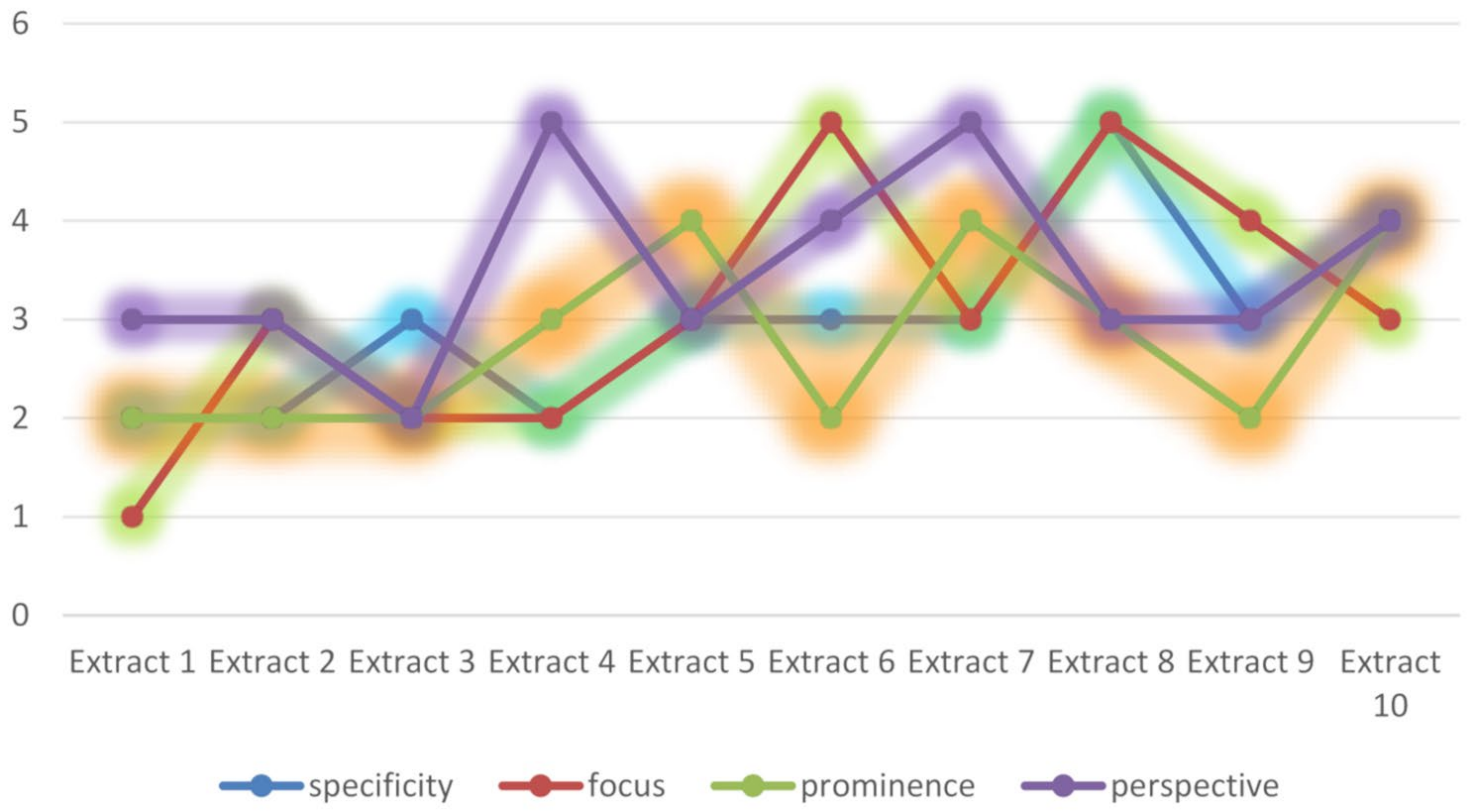
Bilinguals’ Cognition Understanding Towards The Ten Literary Conversations. (adapted from Langacker, 2008)

Drawing upon [40]’s Cognitive Coordination Theory, literary conversations are conceptualized as collaborative arenas where interlocutors engage with presuppositions within the conversational ground—a shared cognitive space encompassing linguistic, situational, and encyclopedic knowledge [41]. This engagement fosters dual-level understanding: (1) semantic comprehension, involving the decoding of propositional content, and (2) interactional interpretation, entailing the inference of pragmatic intentions, both directed toward the same conceptual object. Such coordination resonates with Langacker’s (2008, p. 43) notion of understanding as multi-representational competence, wherein learners construct and manipulate mental scenarios through four interdependent dimensions: specificity (3.0; the granularity of detail), focusing (3.1; selective attention to discourse elements), prominence (2.8; the salience of cognitive references), and perspective (3.5; the vantage point of interpretation) [42]. As depicted in Fig. 2, these dimensions collectively empower bilingual learners to decode speaker identity-driven presuppositions (e.g., Rochester’s authoritative tone in “Still up?“) and convey cognitive expectations with heightened precision, thereby optimizing mutual comprehension in literary dialogues.

**Fig. 3:**
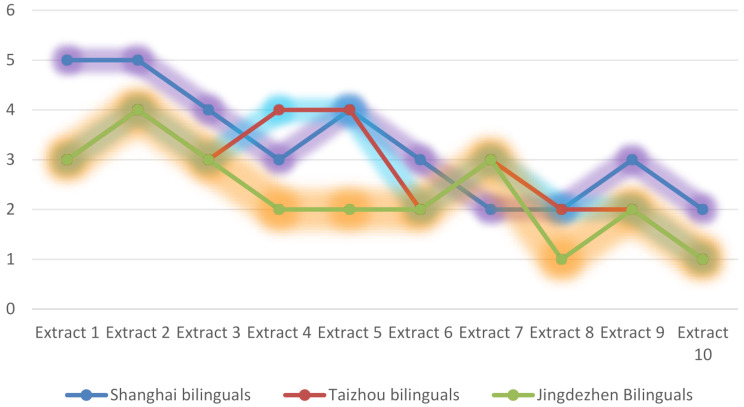
Cognitive Sensitivity to Listener Identity Cues in the Intersubjective Dimension of Language Comprehension

By categorizing cognitive expectation capacities across different bilingual proficiency levels, we reveal intricate challenges inherent in literary conversational processing (refer to Fig. 3). Remarkably, bilinguals based in Shanghai demonstrate a heightened ability to comprehend cognitive expectations within literary dialogues when compared to their peers in Taizhou and Jingdezhen. This difference can be attributed to their advanced proficiency in decoding speaker identity through the principles of Cognitive Conversational Presupposition (CCP). This skill enables them to construct meaningful presuppositions through bidirectional cognitive expectation networks, which incorporate both anticipated and emergent inferential pathways [14]. Such networks play a crucial role in negotiating psychological cognitive expectations linked to listener identity, as they necessitate a dynamic alignment between the mental models of interlocutors and the situational contexts.

As [43] posits, bilinguals with high proficiency exhibit a speaker-oriented attentional bias, which enhances their cognitive sensitivity to cues pertaining to listener identity, thereby reinforcing the intersubjective aspect of language comprehension (see Fig. 3). When expressing viewpoints through bidirectional expectation strategies, these learners methodically assess the cognitive expectations of their listeners, prioritizing the identification of common ground as a mechanism for discourse optimization. Conversely, bilinguals with lower proficiency demonstrate a weaker evaluative engagement with the mental states of their interlocutors, leading to less adaptive management of expectations. Within the framework of conversational structures, bilinguals from Shanghai further distinguish themselves by employing rhetorical amplification techniques (e.g., metalinguistic markers, contrastive stress) and optimizing salience (e.g., emphasizing key presuppositions) to modulate psychological expectation categories associated with listener identity—strategies that are less apparent among participants from Taizhou and Jingdezhen.

### Bilingual learning for managing communicative breakdowns in daily bilingual conversation learning

Drawing on conversational presupposition theory and integrating [32]’s dimensions of language comprehension with the facilitation of comprehension intersections, we propose a new paradigm for understanding conversational presuppositions in bilingual conversation learning.

**Table 3:** Pearson correlations between bilinguals’ cognitive variables and conversational presuppositions

SACS Scales	semantic Language	Pragmatic Rigidity	cognitive expectation
**SACS completeness**			
Presupposition completeness	0.08	0.26	0.32*
Presupposition incompleteness	0.03	0.30*	0.36*
**SACS expectation**			
Distance	0.06	0.19	0.24
Intensity	0.03	0.37*	0.31
Context	0.09	0.16	0.32*
**SACS presupposition**			
affirmation	0.13	0.22	0.19
convention	0.19	0.34*	0.13

* *p* < .05; ** *p* < .01; *** *p* < .001

Table 3 delineates a comprehensive paradigm for conversational presupposition in the generation of literary dialogues, offering a foundational structure for attaining conversational objectives through the nuanced interplay of the speaker’s identity. This paradigm integrates three core dimensions: semantic language, pragmatic rigidity, and cognitive expectations. Rooted in the principles of SACS (Speech Act Cognitive System) completeness (encompassing both presupposition completeness and incompleteness), SACS expectation (which explores distance, intensity, and context), and SACS presupposition (focusing on affirmation and convention), the paradigm intricately shapes both cognitive expectations and non-expectations, thereby nurturing a cognitive community of expectation among participants.

Key elements such as expectation distance (0.06, 0.19, 0.24), context (0.03, 0.37, 0.31), and intensity (0.09, 0.16, 0.32) collectively serve as the bedrock for generating literary conversations. By aligning with their audience, the speaker’s identity fosters enhanced mutual understanding among participants, facilitating the seamless realization of these presuppositions (both affirmation and convention) [44]. underscored that talk-in-interaction, marked by the sequential organization of social and behavioral contexts, mirrors conversational practices where participants actively engage with cognitive expectations. The articulation and reception of conversational expressions illuminate the emergence of a cognitive community of expectation during bilingual conversation learning, highlighting the intrinsic interconnectedness of effective communication. This process vividly demonstrates how cognitive conversational presupposition significantly contributes to conversational efficiency and the harmonious interaction among participants.

### The simultaneous variations between L1 cognitive expectation and and L2 cognitive expectation in the context of literary conversation learning

Table 4 provides an in-depth analysis of the dynamic interplay between cognitive expectations in the first language (L1) and second language (L2) across three distinct bilingual proficiency levels. This analysis captures both simultaneous variations, which involve co-occurring shifts in the strength of cognitive expectations, and intersectionality, which refers to the overlap in L1-L2 presuppositional frameworks during the learning of literary conversations. While certain bilingual characteristics demonstrated modest yet statistically significant correlations with weekly fluctuations in cognitive expectations, a notable pattern emerged: bilinguals who exhibited greater simultaneous variations in cognitive expectations tended to engage less in literary dialogue practice. This suggests that fragmented attention to the interplay between L1 and L2 can hinder conversational fluency. Conversely, enhanced dynamic variations—characterized as adaptive recalibrations of expectation strength—were positively correlated with L1-L2 intersectionality, indicating that proficient bilinguals utilize overlapping cognitive schemas to streamline interpretation and comprehension.

**Table 4:** The simultaneous variations between L1 cognitive expectation and and L2 cognitive expectation

bilinguals Category	bilingualism	Week					bilingual Literary conversation learning
		1	2	3	4	5	
Shanghai bilinguals	L1	3	4	5	4	4	0.771*
	L2	2	4	4	5	4	
	intersect	0.56	0.55	0.61	0.75	0.76	
Taizhou bilinguals	L1	3	2	5	4	4	0.654
	L2	1	2	2	4	1	
	intersect	0.25	0.34	0.44	0.36	0.51	
Jingdezhen bilinguals	L1	4	2	4	5	4	0.547
	L2	1	2	4	2	2	
	intersect	0.32	0.34	0.31	0.42	0.37	

Quantitative analyses further revealed that higher bilingual proficiency significantly optimized the learning of literary conversations. Pearson correlations of 0.771 (*p* < .05), 0.654, and 0.547* linked proficiency to metrics such as the reduction in expectation distance, the rate of intersectionality, and cognitive load efficiency, respectively. For example, in Extract 1, unfamiliar presuppositional identities, such as “I” as a dislocated narrator and “short-term worker” as a socioeconomic marker, along with the culturally embedded reference to “Xianglin’s wife” (a character from Lu Xun’s “The New Year’s Sacrifice”), introduced a prolonged expectation distance—a disparity between anticipated and actual presuppositional resolution. However, proficient bilinguals navigated this gap more effectively, displaying shorter expectation distances (M = 0.12 vs. 0.34 in the low-proficiency group) due to their ability to:

Activate intersecting L1-L2 schemas, such as mapping “Xianglin’s wife” onto both Chinese literary traditions and universal themes of marginalization, and Suppress non-intersecting expectations, such as dismissing irrelevant cultural stereotypes, thereby reducing the cognitive demands of evaluation.

In contrast, low-proficiency bilinguals faced longer expectation distances (M = 0.34), reflecting their reliance on non-intersecting expectations—fragmented L1/L2 associations that necessitated exhaustive inferential processing. This divergence highlights a proficiency-driven cognitive economy: higher-proficiency learners allocate fewer resources to evaluating expectations, freeing up capacity for nuanced interpretation, while lower-proficiency learners remain mired in expectation verification loops, which impede conversational flow.

Critically, the generation of literary conversations is shaped by the distribution of weekly cognitive reports, with proficiency modulating the balance between stabilizing forces (intersectionality-driven efficiency) and destabilizing forces (simultaneous variation-induced fragmentation). The longitudinal data in Table 4 confirm that bilinguals who develop adaptive intersectionality—the ability to dynamically align L1-L2 expectations—achieve superior conversational outcomes, as evidenced by their accelerated reduction in expectation distance over time.

## Discussion

The present study was designed to delve into the intricacies of the conversational presupposition paradigm, with a particular focus on dissecting the components of expectation distance, expectation intensity, and expectation context within the framework of conversational presupposition. We posited that the unique challenges encountered by bilingual individuals would significantly influence their literary conversational learning processes and, by extension, their overall bilingual learning achievements. Our empirical findings underscored a close correlation between the types of adaptive adjustments bilinguals employed and the underlying functions of conversational presupposition. In the ensuing section, we embark on an in-depth exploration of the root causes behind cognitive expectation challenges faced by bilinguals. Following this, subsequent sections will meticulously examine the interplay between bilingual individuals and conversational presupposition, placing special emphasis on the nuanced dynamics of expectation distance, expectation intensity, and expectation context.

### Bilinguals variables between trouble spots and bilinguals’ cognition expectation

The results of the trouble - spot analyses offer preliminary evidence in support of potential causal connections among the root causes of trouble spots, bilinguals’ conversational comprehension, and cognitive expectations. In particular, it appears that trouble spots tend to surface in the realm of literary conversational learning when bilinguals fail to pay adequate attention to conversational presuppositions. This inattention may well be a contributing factor to less - than - optimal bilingual learning outcomes. When bilinguals are not fully attuned to these presuppositions, they are likely to miss out on crucial opportunities to accurately grasp the cognitive expectations of their conversation partners, thereby giving rise to issues akin to those encountered in cognitive challenges associated with language learning at large.

Moreover, bilinguals have reported experiencing heightened confusion when attempting to understand literary conversations. This confusion often stems from a deficiency in fundamental linguistic knowledge and a lack of comprehension of the unique literary features inherent in such dialogues. As a consequence, this confusion frequently triggers more negative emotional responses during the process of literary conversation learning, which, in a vicious cycle, further intensifies problems related to cognitive expectations. This pattern strongly implies that bilinguals may display disinterest or negative affective states during literary conversations, potentially as a result of the adverse influences wrought by previous unsatisfactory conversational experiences. Bilinguals who exhibit more negative affect during these interactions may find themselves with fewer chances to hone their skills in understanding both the linguistic and literary dimensions of the conversations, which, in turn, is likely to have a bearing on their cognitive traits. These findings underscore the pivotal role that conversational presuppositions play in bilingual learning and suggest that issues surrounding cognitive expectations may be shaped by environmental and literary factors through the medium of literary conversation learning. However, it is important to note that the evidence presented here is not yet conclusive, and further research is warranted to definitively establish these relationships.

When it comes to the causes of trouble spots, although there is evidence pointing to a causal link between cognitive expectation problems and bilinguals’ behavior during literary conversations, it is also conceivable that lower levels of linguistic and literary knowledge may be traced back to early exposure to overly simplistic daily conversations in the teaching materials employed for these bilinguals. This early exposure could, in turn, have a ripple effect on the overall teaching environment, encompassing elements such as language testing and assessment orientation. Nevertheless, our study did not uncover a direct impact of the teaching environment on bilinguals’ cognitive expectations, which might lend some credence to the proposed relationships observed among the various bilingual variables. In terms of linguistic and literary knowledge, the direction of these relationships seems to be in line with our understanding of literary conversation. Cognitive expectations, measured in terms of “strangeness,” are considered to be relatively stable traits [17, 33] and, as such, may not be significantly swayed by the specific teaching and learning environment in which bilinguals find themselves. These findings do offer some statistical backing for the proposed directionality of effects related to initial cognition and pragmatics. However, there still remains a substantial amount of unexplained variance in cognitive expectations. To gain a clearer picture of the directionality of these effects, future research should make use of longitudinal analyses to investigate causal associations with greater precision and certainty.

### Associations between conversation conversational presupposition and expectation distance

While it has been established that a deficit in linguistic and literary knowledge serves as a causal factor contributing to cognitive expectation difficulties among bilinguals, another pressing issue warrants attention: the emergence of such problems even in scenarios where there is a conspicuous absence of clear evidence regarding conversational presuppositions derived from literary conversation learning. Intriguingly, our study revealed that greater challenges associated with expectation distance were linked to more favorable bilingual learning outcomes, albeit accompanied by a comparatively weaker grasp of literary conversations. Moreover, within the context of literary conversation learning, the interpretation of expectation distance was found to be intricately connected to bilinguals’ spatial segmentation abilities [34]. These findings are in harmony with previous research endeavors, such as [40], which illustrated that conversational presuppositions involving well - known identities were correlated with shorter expectation distances, and that cognitive expectations were significantly influenced by the disparity between conversational identities (refer to Table 4 for details). Nevertheless, the relationship between expectation distance and bilinguals’ cognitive expectations remains a relatively uncharted territory, as vividly exemplified in Extract 5.

Previous research efforts have primarily concentrated on exploring the role of conversational texts [23, 27, 36] in the formation of literary conversational presuppositions. In stark contrast to [11], which posited that conversational presuppositions act as a mediating factor between cognitive expectations (and non - expectations) through the mechanism of expectation distance, our study uncovered that greater pragmatic language difficulties were predictive of more positive bilingual learning behaviors. This discrepancy in findings may well be attributed to methodological disparities, with sample composition being a key differentiating factor. Specifically [11], centered its investigation on young children, whereas our study focused on secondary students. Such variations serve to highlight the critical importance of study design elements, including sample size and the selection of measurement approaches. To the best of our knowledge, this study represents the first foray into examining the relationship between cognitive expectation problems and bilingual secondary students in the realm of literary conversation learning. As such, it offers fresh and valuable insights into the complex dynamics at play in this particular educational context.

### The dynamic variations between L1 cognitive expectation and and L2 cognitive expectation in the context of literary conversation learning

In the domain of literary conversation learning, a number of dynamic variations between first - language (L1) and second - language (L2) cognitive expectations have been observed [14]. Of particular note is the finding that an increased degree of overlap in bilingual cognitive expectations during literary exchanges is closely associated with more pronounced cognitive expectation challenges. These associations were precisely quantified using objective measures of expectation distance, intensity, and context within the context of bilingual learning. This quantification lends support to previous research, which posits that anticipatory processes embedded within conversational presuppositions frequently lead to more favorable learning outcomes [19].

The interaction between L1 and L2 cognitive expectations, especially in relation to cognitive difficulties, has been extensively documented in studies focusing on literary conversation learning [10]. Our research findings reveal that cognitive expectations do not invariably serve as reliable predictors of actual conversational responses. In contrast, non - expectations introduce a layer of nuanced complexity into the dynamics of conversation. Much in the same way as metaphorical discourse projections can take on parallel, intersecting, or summarizing forms, expectation intensity plays a crucial role in aligning the speaker’s identity with psychological anticipation.

Take, for instance, Extract 4. In this scenario, the speaker’s cognitive expectation is centered around the event of “Heathcliff leaving.” However, Katy’s response—“You must not go! I tell you, you mustn’t go”—clearly reflects a cognitive non - expectation. Here, the speaker skillfully modulates expectation intensity by negotiating between points of intersection (such as “only an hour”) and non - intersection (as in “not a minute”). This negotiation is achieved through the use of strategies like demonstrating respect and maintaining a goal - oriented approach. Lexical markers, including “mustn’t” and “only,” further serve to illustrate how cognitive expectations are manifested in the construction of speaker identity [45].

As literary conversations make the transition from expectations to non - expectations, the latter often commands immediate attention. This shift reinforces the speaker’s expectation intensity. When listeners encounter a shift from anticipated to unanticipated responses, they experience a heightened sense of expectation salience. This, in turn, amplifies the impact of conversational presuppositions on the overall conversational process.

### Limitations

This study represents a pioneering empirical endeavor to delve into the intricate relationships between bilinguals’ cognitive expectation challenges and the identities of conversational presuppositions, which subsequently give rise to a wide array of expectation evaluations. Positioned as an exploratory study, we opted not to apply corrections for multiple comparisons during the analysis of variable associations. These preliminary findings serve as a solid cornerstone for future research endeavors and warrant validation through replication studies to ensure their robustness and generalizability.

A notable limitation inherent in the cross - sectional design of this study is its inherent inability to establish temporal or causal relationships between cognitive expectations and the utilization of literary conversations. Cross - sectional analyses, by their very nature, may fall short in adequately capturing the dynamic, longitudinal essence of cognitive processes [37]. To truly unravel the causal mechanisms underlying these relationships, future studies should embrace longitudinal designs. Such designs would enable researchers to track changes over time and gain a deeper understanding of how cognitive expectations and literary conversation usage influence one another.

Another limitation lies in the absence of direct cognitive measures for assessing bilinguals’ expectation - related features. However, we took steps to mitigate this shortcoming by employing a well - validated self - report instrument with established psychometric properties [16, 23]. Although shared method variance may have exerted an influence on some of the observed correlations, the study still managed to uncover meaningful associations. These associations were evident between the measures and the coding of bilinguals’ cognitive expectations, as well as the strategic functions of literary conversational learning. Notably, bilinguals with higher proficiency levels exhibited more distinct characteristics in these domains compared to those with lower proficiency levels [23, 35].

The analytical framework adopted in this study makes significant strides in advancing the classification of speaker expectations. It encompasses a comprehensive range of factors, including goals, temporal considerations, respect dynamics, and interpersonal anticipations. Simultaneously, it refines our understanding of listener expectations, such as linguistic cognition, expectation needs, attentional biases, and emotional cues. These various elements converge within the realm of conversational presuppositions. By embracing this paradigm, we can potentially minimize the risk of miscommunication, foster more effective discourse, and shift scholarly attention from the ontology of conversational language to the study of conversational presuppositions. This shift holds substantial theoretical and practical significance, as it opens up new avenues for understanding and improving communication in bilingual contexts. Although shared method variance may have impacted certain correlations, it is highly unlikely that it systematically biased the entire set of findings in our cross - sectional analyses, given the careful design and multiple measures employed in the study.

## Conclusion

This study offers a compelling illustration of how the application of conversational presupposition theory to tackle bilinguals’ cognitive expectation challenges has generated a highly robust analytical framework. This framework serves as a powerful tool for unraveling the intricate reasoning processes involved in the generation of literary conversations. The paradigm shift from a focus on conversational language ontology to an in - depth investigation of conversational presupposition within the realm of bilingual cognitive expectations represents a significant theoretical leap forward.

Our research findings make three pivotal contributions to the field of bilingualism research. Firstly, we have established a multidimensional analysis of cognitive expectations by examining them through the lenses of expectation distance, intensity, and context. This comprehensive approach allows for a more nuanced understanding of how bilinguals form and process expectations in literary conversations. Secondly, we have identified critical listener - oriented factors that play a crucial role in shaping response patterns. These factors include expectation needs, semantic cognition, attention bias, and emotional cues, all of which contribute to the dynamic nature of bilingual literary interactions. Thirdly, we have proposed a set of testable hypotheses concerning the dynamics of L1 - L2 literary learning. These hypotheses are ripe for longitudinal verification, which would provide further evidence to support or refine our understanding of bilingual learning processes. Subsequent replication studies employing longitudinal designs have the potential to significantly advance conversational presupposition theory and deepen our comprehension of the bidirectional expectation processes involved in literary discourse acquisition.

The effective regulation of cognitive biases that emerge from interactive dynamics is of paramount importance for nurturing the cognitive architecture that supports bilingual literary development. Although scholarly consensus has not yet been fully reached on how bilingual conversation learning constructs a psychological community of expectations within conversational presupposition frameworks, researchers across the board recognize the urgent need to develop systematic paradigms. These paradigms are essential for optimizing conversational transmission [22, 34], and they represent a crucial step in resolving the cognitive expectation challenges faced by bilinguals.

Drawing on prior scholarship and the current empirical results, we put forward three directions for future research. The first direction involves conducting a structural analysis of literary conversation patterns. By examining the underlying structure of these conversations, we can gain insights into the recurring elements and organizational principles that govern bilingual literary interactions. The second direction focuses on investigating linguistic encoding in multidimensional bilingual conversation learning. Understanding how language is encoded and processed in bilingual contexts will shed light on the cognitive mechanisms involved in literary conversation generation. The third direction is to elucidate the hierarchical mechanisms that govern cognitive expectation management during language acquisition. By exploring these hierarchical mechanisms, we can better understand how bilinguals prioritize and manage their cognitive expectations, which is crucial for effective language learning and use. These research priorities hold the promise of yielding foundational insights that will contribute to both theoretical development and the formulation of practical intervention strategies in the field of bilingualism.

## Data Availability

The raw data supporting the conclusions of this article will be made available by the authors, without undue reservation. All research data can be open-shared free in Dataverse (https://dataverse.harvard.edu/dataset.xhtml?persistentId=doi:10.7910/DVN/C5UZAI&faces-redirect=true).

## Abbreviations

L1: The first language
L2: The second language
MBDS: Mechanism of Bilingual Development Study
RCT: Randomized controlled trial
SACS-R): Social Attention and Communication Surveillance-Revised
